# Circumferential wall enhancement with contrast ratio measurement in unruptured intracranial aneurysm for aneurysm instability

**DOI:** 10.1002/brb3.2568

**Published:** 2022-04-05

**Authors:** Xiao‐Bing Wu, Jing‐Lian Zhong, Sheng‐Wen Wang, Yun Su, Pei‐Sheng Chen, Zhong‐Jun Li, Chun Xiang, Wang‐Qing Cai, Zhong‐Song Shi

**Affiliations:** ^1^ Department of Neurosurgery Sun Yat‐sen Memorial Hospital Sun Yat‐sen University Guangzhou China; ^2^ Department of Radiology Sun Yat‐sen Memorial Hospital Sun Yat‐sen University Guangzhou China

**Keywords:** aneurysm wall enhancement, ELAPSS score, PHASES score, unruptured intracranial aneurysm, vessel wall imaging

## Abstract

**Background:**

Aneurysm wall enhancement on high‐resolution vessel wall imaging (HR‐VWI) may represent vessel wall inflammation for unruptured intracranial aneurysms (UIAs). Further evidence for the role of circumferential aneurysm wall enhancement (CAWE) in evaluating the instability of UIAs is required, especially in small aneurysms (<7 mm).

**Methods:**

We analyzed patients with saccular UIAs who prospectively underwent HR‐VWI on a 3.0 T MRI scanner in our center from September 2017 to August 2021. The presence of AWE was identified and quantitatively measured using the aneurysm‐to‐pituitary stalk contrast ratio (CRstalk) with maximal signal intensity value. The PHASES and ELAPSS scores were used to assess the risk of aneurysm rupture and growth. We evaluated the association of CAWE and CRstalk value with intracranial aneurysm instability.

**Results:**

One hundred patients with 109 saccular UIAs were included in this study. Eighty‐three UIAs (76.1%) had a size smaller than 7 mm. PHASES and ELAPSS scores were significantly higher in UIAs with CAWE than in UIAs without CAWE (*p* < .01). The association of CAWE with PHASES and ELAPSS scores remained in small UIAs (<7 mm). The optimal cutoff value of CRstalk for CAWE was 0.5. PHASES and ELAPSS scores were significantly higher in UIAs with CRstalk ≥0.5 than in UIAs with CRstalk <0.5 (*p* < .01).

**Conclusions:**

CAWE on HR‐VWI is a valuable imaging marker for aneurysm instability in UIAs. CRstalk value ≥0.5 may be associated with a higher risk of intracranial aneurysm rupture and growth.

## Graphical abstract

Circumferential aneurysm wall enhancement (CAWE) on high‐resolution vessel wall imaging (HR‐VWI) is a valuable imaging marker for aneurysm instability in small unruptured intracranial aneurysms. The value of aneurysm‐to‐pituitary stalk contrast ratio (CRstalk) ≥0.5 may be associated with a higher risk of intracranial aneurysm rupture and growth.

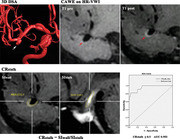

## 1 INTRODUCTION

Unruptured intracranial aneurysms (UIAs) are pathological dilatations of the intracranial arteries with a population prevalence of approximately 3.2%, mainly in the circle of Willis and intracranial arterial bifurcations (Thompson et al., 2015). With the development of neuroimaging technology, UIAs have been discovered with increasing frequency. The rupture risk of UIAs is low, with an annual rupture rate of approximately 1%. UIAs are the leading cause of spontaneous subarachnoid hemorrhage and can result in severe consequences once ruptured (Thompson et al., 2015). Therefore, early and effective identification of UIAs with a high risk of rupture is crucial. The need for the early treatment of UIA is currently determined in clinical practice mainly based on aneurysm size, morphology, and location. Surgical clipping or endovascular treatment has been recommended for aneurysms larger than 7 mm and those in the posterior circulation site (Wiebers, 2003). However, management options for UIAs smaller than 7 mm are unclear.

Vessel inflammation has been shown to play an important role in the occurrence, development, and rupture of UIAs (Labeyrie et al., 2017; Tulamo et al., 2018). With the development of neuroimaging techniques, high‐resolution vessel wall imaging (HR‐VWI) on magnetic resonance can reveal aneurysm wall enhancement (AWE), reflecting the inflammatory status of UIAs (Edjlali et al., 2014; Hudson et al., 2019; Matouk et al., 2013). The assessment of AWE in UIAs mainly adopted a qualitative method, which had the advantages of simplicity and easy operation but could not avoid the subjectivity of the readers. Quantitative assessment of AWE has been recently proposed to analyze the degree of wall enhancement in UIAs, reducing the personal misjudgment of readers (Omodaka et al., 2019, 2016; Roa, Zanaty, Osorno‐Cruz, et al., 2020). Although AWE on HR‐VWI may represent vessel wall inflammation for UIAs, further evidence for the role of AWE in evaluating the risk of rupture and growth of UIAs is required, especially in small aneurysms (<7 mm). The purpose of this study was to investigate the association of aneurysm wall enhancement with the risk of rupture and growth in UIAs.

## 2 MATERIALS AND METHODS

### 2.1 Study population

One hundred fifty‐two consecutive patients with 162 UIAs were identified from a prospectively maintained database between September 2017 and August 2021 at our institution. The local institutional review board approved this study at our institution. The inclusion criteria were as follows: (1) age more than 18 years; (2) no clear history of subarachnoid hemorrhage; (3) untreated saccular UIAs confirmed by digital subtraction angiography; (4) aneurysm identified on MR angiography and images of HR‐VWI without artifacts. We exclude the data of UIAs in the extracranial or cavernous sinus of the internal carotid artery (ICA).

We acquired data, including demographic characteristics, vascular risk factors, and the number, location, size, and morphology of UIAs. The PHASES and ELAPSS scores were used to assess the rupture and growth risk of UIA, which represented the intracranial aneurysm instability (Backes et al., 2017; Greving et al., 2014).

### 2.2 Aneurysm wall enhancement on HR‐VWI

MR was performed on a 3.0T MRI scanner (Achieva TX, Philips Healthcare, Best, the Netherlands) with a 32‐channel head coil. The HR‐VWI protocol included a two‐dimensional (2D) T1‐weighted black‐blood vessel wall sequence and a 3D T1‐weighted volume isotropic turbo‐spin‐echo acquisition (VISTA) sequence before and after intravenous injection of 0.1 mmol/kg gadopentetate glucosamine (Gd‐DTPA, Hokuriku Pharmaceutical, China). Patients with UIAs were scanned with the 2D high‐resolution T1‐weighted sequence between September 2017 and September 2018 and scanned with the 2D high‐resolution T1‐weighted VISTA sequence since October 2018.

The imaging parameters of 2D T1‐weighted black‐blood were as follows: TR/TE = 1000 ms/9 ms; field of view = 150 mm × 150 mm; matrix = 332 × 269; number of layers = 5; resolution = 0.45 mm × 0.55 mm; layer thickness = 3 mm, layer spacing = 0, flip angle = 80°; bandwidth = 300 Hz/pixel; the total scan time = 3 min and 45 s. The imaging parameters of 3D T1 VISTA were as follows: TR/TE = 700 ms/35 ms; field of view = 200 mm × 251 mm; matrix = 252 × 314; spatial resolution = 0.8 mm × 0.8 mm × 0.8 mm; layer number = 171; layer spacing = −0.4 mm; flip angle = 80°; bandwidth = 300 Hz/pixel; the total scan time = 4 min and 55 s. The BB pulse with motion‐sensitized driven‐equilibrium (MSDE) was used in this study. MSDE applies a magnetization preparation sequence that causes moving spins to diphase and thereby suppresses signals from blood vessels with sufficient flow.

**FIGURE 1:**
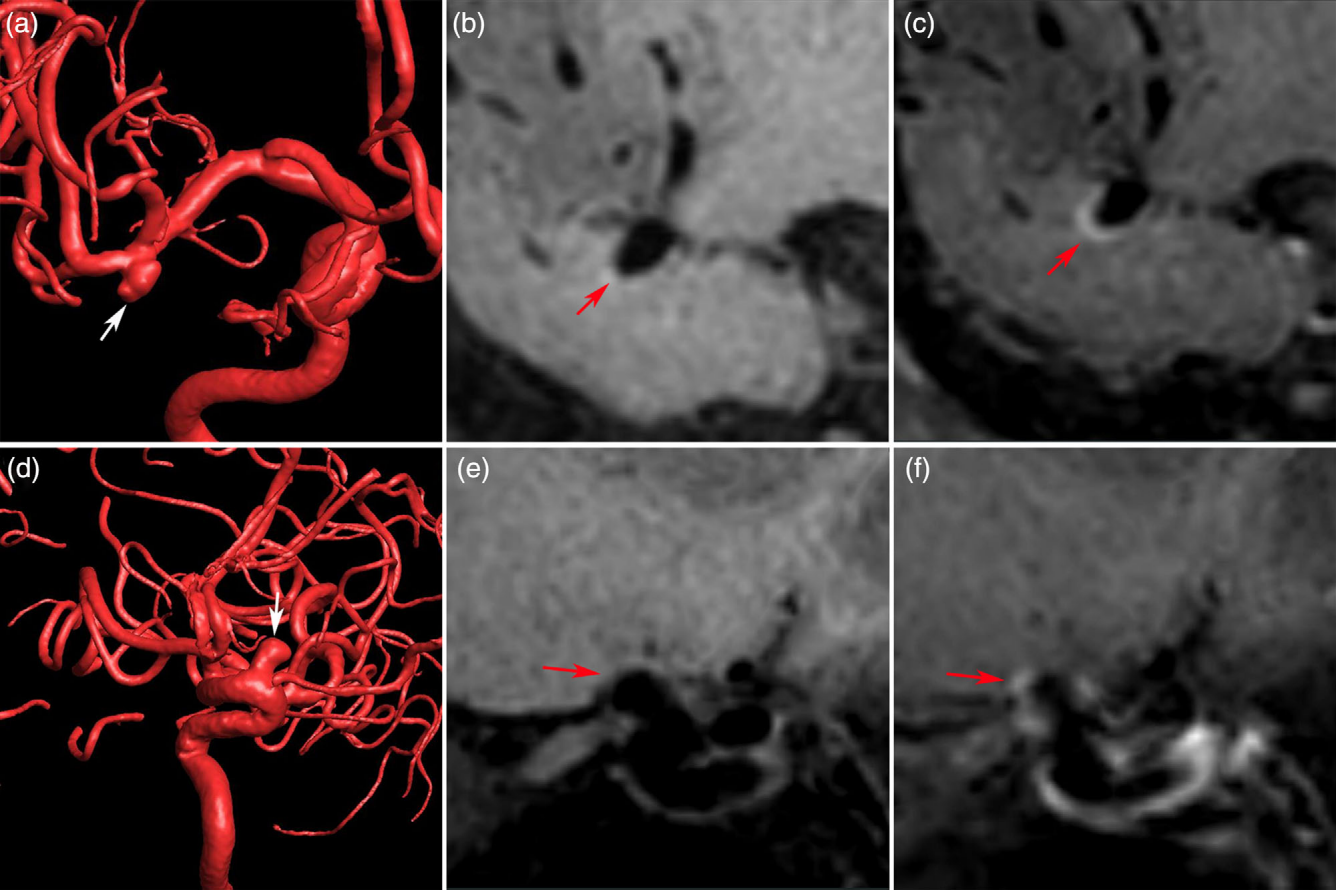
Circumferential aneurysm wall enhancement on high‐resolution vessel wall imaging (HR‐VWI) is shown in a middle cerebral artery aneurysm (a, three‐dimensional (3D) digital subtraction angiography; b, precontrast 3D HR‐VWI; c, postcontrast 3D HR‐VWI). Focal aneurysm wall enhancement on HR‐VWI is shown in an internal carotid artery aneurysm (d, 3D digital subtraction angiography; e, precontrast 3D HR‐VWI; f, postcontrast 3D HR‐VWI)

**FIGURE 2:**
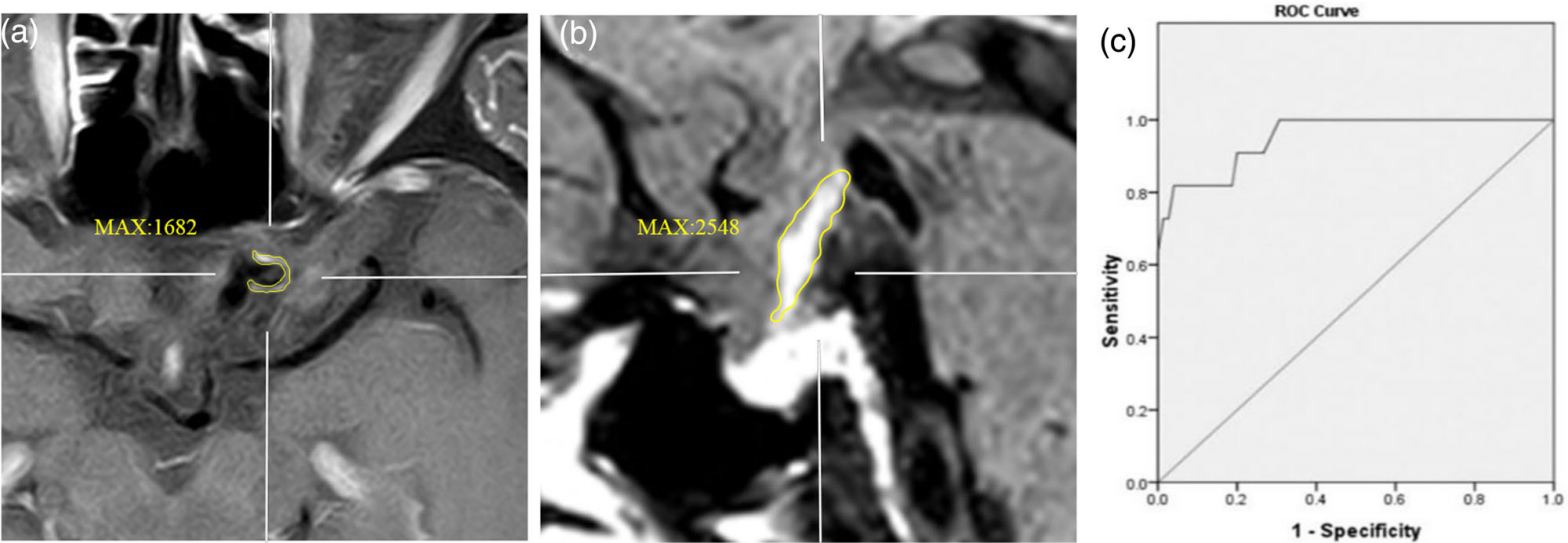
Example of aneurysm‐to‐pituitary stalk contrast ratio (CRstalk) measurement in an internal carotid artery (ICA) aneurysm. The maximal signal intensity value of aneurysm wall and pituitary stalk on postcontrast T1‐weighted images was used to calculate the CRstalk (CRstalk = SIwall/SIstalk). Postcontrast T1‐weighted images of left ICA aneurysm (a) and sagittal images of pituitary stalks (b) showing each maximal signal intensity (MAX). CRstalk of the aneurysm was calculated as 0.66. Receiver operating characteristic curve of CRstalk to differentiate circumferential aneurysm wall enhancement, the area under the curve was 0.953 (c)

Contrast enhancement of UIAs was qualitatively classified into none of AWE, focal AWE, and circumferential AWE (CAWE) according to the pre‐ and postcontrast HR‐VWI T1‐weighted sequences (Figure 1) (Hu et al., 2016; Xiao et al., 2018). Focal AWE involved the neck, the dome, the intermediate portion, or a bleb, whereas CAWE involved the entire aneurysm (Figure 1) (Edjlali et al., 2018; Fu et al., 2021). Signal intensity (SI) of the aneurysm wall (SIwall) and the pituitary stalk (SIstalk) on the 3D postcontrast HR‐VWI T1‐weighted sequence were measured. The maximal SI value of SIwall and SIstalk was used to calculate the aneurysm‐to‐pituitary stalk contrast ratio (CRstalk) as CRstalk = SIwall/SIstalk. CRstalk using maximal SI value was an optimal quantitative method for the analysis of UIA wall enhancement (Figure 2) (Omodaka et al., 2019, 2016; Roa, Zanaty, Osorno‐Cruz, et al., 2020).

### 2.3 Statistical analysis

Two readers who were blinded to the clinical data independently reviewed the HR‐VWI T1‐weighted images to identify the presence and pattern of AWE. A third reader resolved disagreements. Cohen *κ* statistics were used to assess the interreader agreement. *κ* Values > 0.80 were regarded as excellent for the identification of AWE (Landis & Koch, 1977). The cutoff values for CRstalk with the best sensitivity and specificity to differentiate CAWE from non‐CAWE were identified by analyzing the receiver operating characteristic (ROC) curve.

We analyzed the relationships among clinical data, aneurysm characteristics, PHASES score, and ELAPSS score with CAWE and CRstalk in all UIAs and the subgroup of aneurysms smaller than 7 mm. We used SPSS 22.0 software for statistical analysis. Student's *t*‐test or Mann–Whitney *U* test was used for continuous variables. Fisher's exact or chi‐square test was used for the categorical variables. *p*‐Value < .05 was considered statistically significant for the results.

## 3 RESULTS

### 3.1 Clinical characteristics

One hundred patients with 109 saccular UIAs were included in this study. Fifty‐two patients were excluded due to fusiform aneurysm (*n* = 28), extracranial or cavernous sinus of the ICA aneurysm (*n* = 7), without digital subtraction angiography examination (*n* = 5), aneurysm not been identified on MR angiography or with unfavorable HR‐VWI images (*n* = 12). The mean age was 56.7 ± 11.2 years, and 60 (60%) were women. The mean size of aneurysms was 5.9 ± 4.6 mm (1.3–37.8 mm), with 36 (33%) presented with irregular shapes. Eighty‐three UIAs (76.1%) had a size smaller than 7 mm. Sixty‐five (59.6%) aneurysms located in ICA, 19 (17.4%) aneurysms in middle cerebral artery, five (4.6%) aneurysms in posterior circulation, nine (8.2%) in posterior communicating artery, and 11 (10.1%) in anterior cerebral artery. The mean point of PHASES and ELAPSS scores was 3.0 ± 2.9 and 11.4 ± 7.0, respectively. The characteristics of the patients and aneurysms are shown in Table 1.

**TABLE 1:** Characteristics of unruptured intracranial aneurysms with and without circumferential aneurysm wall enhancement

	Total	CAWE	Non‐CAWE	
	(*n* = 109)	(*n* = 21)	(*n* = 88)	*p*‐Value
Age (yr)	56.7 ± 11.2	56.9 ± 14.3	56.7 ± 10.3	.951
Female	60 (60%)	10 (47.6%)	50 (63.3%)	.193
Hypertension	59 (59%)	15 (71.4%)	41 (51.9%)	.109
Diabetes	12 (12%)	1 (4.8%)	11 (13.9%)	.451
Smoking	25 (25%)	6 (28.6%)	19 (24.1%)	.671
Drinking	5 (5%)	2 (9.5%)	3 (3.8%)	.282
Size (mm)	5.9 ± 4.6	10.20 ± 8.08	4.93 ± 2.36	.007
0.0–6.9	83 (76.1%)	8 (38.1%)	75 (85.2%)	<.001
≥7.0	26 (23.9%)	13 (61.9%)	13 (14.8%)	
Aspect ratio	1.29 ± 0.80	2.02 ± 1.45	1.11 ± 0.40	.01
Size ratio	1.95 ± 1.41	3.34 ± 2.14	1.62 ± 0.91	.002
Irregular shape	36 (33.0%)	11 (52.4%)	25 (28.4%)	.036
Location				.073
ICA	65 (59.6%)	8 (38.1%)	57 (64.8%)	
MCA	19 (17.4%)	5 (23.8%)	14 (15.9%)	
ACA/PComA/PC	25 (22.9%)	8 (38.1%)	17 (19.3%)	
PHASES score	3.0 ± 2.9	6.21 ± 2.97	2.24 ± 2.31	<.001
0–4	79 (72.5%)	9 (42.9%)	70 (79.5%)	<.001
5–7	22 (20.2%)	6 (28.6%)	16 (18.2%)	
8–9	4 (3.7%)	2 (9.5%)	2 (2.3%)	
≥10	4 (3.7%)	4 (19.0%)	0	
ELAPSS score	11.4 ± 7.0	18.05 ± 6.05	9.32 ± 5.86	<.001
0–9	48 (44.0%)	3 (14.3%)	45 (51.1%)	.001
10–19	44 (40.4%)	10 (47.6%)	34 (38.6%)	
≥20	17 (15.6%)	8 (38.1%)	9 (10.2%)	

*Note*: Two morphologic parameters were calculated as the aspect ratio = dome height/neck diameter, size ratio = size/average parent vessel diameter.

Abbreviations: ACA, anterior cerebral artery; CAWE, circumferential aneurysm wall enhancement; ICA, internal carotid artery; MCA, middle cerebral artery; PC, posterior circulation; PComA, posterior communicating artery.

### 3.2 CAWE and aneurysm instability

Twenty‐one patients with twenty‐three UIAs received a 2D HR‐VWI sequence, and 79 patients with 86 UIAs were scanned by a 3D HR‐VWI sequence. AWE on HR‐VWI was present in 40 (36.7%) UIAs, including 19 UIAs with focal AWE and 21 UIAs with CAWE. The interreader agreement for the identification of AWE was excellent, with *κ* = 0.85. The aneurysm size was significantly larger in the CAWE group than in the non‐CAWE group (10.2 mm vs. 4.9 mm, *p* = .007). The proportion of CAWE was significantly lower in the group with aneurysms smaller than 7 mm than those larger than 7 mm (9.6% [eight of 83 patients] vs. 50.0% [13 of 26 patients], *p* < .001). The proportion of irregular shape was significantly higher in the group with CAWE than without CAWE (52.4% vs. 28.4%, *p* = .036). PHASES and ELAPSS scores were significantly higher in the CAWE group than in the non‐CAWE group (6.2 vs. 2.2 points, *p* < .001; 18.1 vs. 9.3 points, *p* < .001, respectively). The proportion of CAWE increased significantly with the increasing PHASES and ELAPSS scores (*p* < .001, *p* < .001, respectively).

In the subgroup with UIAs smaller than 7 mm, PHASES and ELAPSS scores were significantly higher in the CAWE group than in the non‐CAWE group (3.6 vs. 1.7 points, *p* = .007; 12.3 vs. 8.1 points, *p* = .010, respectively) (Table 2). The percentage of CAWE in ICA, middle cerebral artery, and anterior cerebral artery/posterior communicating artery/posterior circulation was 3.9%, 21.4%, and 16.7%, respectively. Compared with the aneurysms located in the ICA, those located in the non‐ICA tended more proportion of CAWE (18.8% [six of 31 patients] vs. 3.9% [two of 51 patients], *p* = .05).

**TABLE 2:** Characteristics of unruptured intracranial aneurysms smaller than 7 mm with and without circumferential aneurysm wall enhancement

	Total	CAWE	Non‐CAWE(*n*	*p*‐Value
	(*n* = 83)	(*n* = 8)	= 75)	
Age (yr)	57.34 ± 10.83	66.50 ± 8.38	56.23 ± 10.60	.010
Female	47 (63.5%)	4 (50%)	43 (65.2%)	.452
Hypertension	41 (55.4%)	6 (75%)	35 (53.0%)	.238
Diabetes	12 (16.2%)	1 (12.5%)	11 (16.7%)	1.000
Smoking	19 (25.7%)	2 (25%)	17 (25.8%)	1.000
Drinking	3 (4.1%)	1 (12.5%)	2 (3%)	.294
Size (mm)	4.20 ± 1.25	4.69 ± 1.20	4.14 ± 1.25	.242
1.0–2.9	13 (15.7%)	0	13 (17.3%)	.402
3.0–4.9	48 (57.8%)	5 (62.5%)	43 (57.3%)	
5.0–6.9	22 (26.5%)	3 (37.5%)	19 (25.3%)	
Aspect ratio	10.5 ± 0.36	0.94 ± 0.29	1.06 ± 0.37	.380
Size ratio	1.42 ± 0.63	1.84 ± 0.69	1.37 ± 0.61	.045
Irregular shape	24 (28.9%)	5 (62.5%)	19 (25.3%)	.041
Location				.075
ICA	51 (61.4%)	2 (25%)	49 (65.3%)	
MCA	14 (16.9%)	3 (37.5%)	11 (14.7%)	
ACA/PComA/PC	18 (21.7%)	3 (37.5%)	15 (20%)	
PHASES score	1.90 ± 1.93	3.63 ± 1.77	1.72 ± 1.86	.007
ELAPSS score	8.46 ± 4.44	12.25 ± 2.92	8.05 ± 4.39	.010

Abbreviations: ACA, anterior cerebral artery; CAWE, circumferential aneurysm wall enhancement; ICA, internal carotid artery; MCA, middle cerebral artery; PC, posterior circulation; PComA, posterior communicating artery.

### 3.3 CRstalk and aneurysm instability

Seventy‐nine patients with 86 UIAs were scanned by a 3D HR‐VWI sequence to quantify the CRstalk values in this study. On ROC curve analysis, the optimal cutoff value of CRstalk to differentiate CAWE from non‐CAWE was 0.5, and the area under the curve was 0.953 (Figure 2). Using a cutoff value of 0.5 for CRstalk, the sensitivity and specificity were 0.818 and 0.960, respectively.

CRstalk values increased significantly with the increasing aneurysm size, PHASES score, and ELAPSS score. The aneurysm size was significantly larger in the group with CRstalk ≥0.5 than those with CRstalk <0.5 (9.2 mm vs. 4.9 mm, *p* = .032). PHASES and ELAPSS scores were significantly higher in the CRstalk ≥0.5 group than in the CRstalk <0.5 group (4.4 vs. 2.2 points, *p* = .006; 16.5 vs. 10.2 points, *p* = .002, respectively). The proportion of CRstalk ≥ 0.5 increased with the increasing PHASES and ELAPSS scores (Table 3).

**TABLE 3:** Characteristics of unruptured intracranial aneurysms with CRstalk ≥0.5 and CRstalk <0.5

	Total	CRstalk ≥0.5	CRstalk <0.5	
	(*n* = 86)	(*n* = 12)	(*n* = 74)	*p*‐Value
Age (yr)	57.23 ± 10.89	59.08 ± 14.62	56.90 ± 10.19	.525
Female	45 (57.0%)	4 (33.3%)	41 (61.2%)	.112
Hypertension	44 (55.7%)	8 (66.7%)	36 (53.7%)	.406
Diabetes	10 (12.7%)	0	10 (14.9%)	.345
Smoking	20 (25.3%)	5 (40%)	15 (22.4%)	.157
Drinking	2 (2.5%)	0	2 (3.0%)	1.000
Size (mm)	5.52 ± 3.31	9.18 ± 6.00	4.92 ± 2.18	.032
0.0−6.9	68 (79.1%)	6 (50%)	62 (83.8%)	.008
≥7.0	18 (20.9%)	6 (50%)	12 (16.2%)	
Aspect ratio	1.22 ± 0.55	1.80 ± 0.95	1.13 ± 0.40	.034
Size ratio	1.78 ± 1.09	2.74 ± 1.46	1.62 ± 0.94	.001
Irregular shape	31 (36.0%)	6 (50%)	25 (33.8%)	.278
Location				.756
ICA	56 (65.1%)	7 (58.3%)	49 (66.2%)	
MCA	15 (17.4%)	3 (25%)	12 (16.2%)	
ACA/PComA/PC	15 (17.4%)	2 (16.7%)	13 (17.6%)	
PHASES score	2.52 ± 2.62	4.42 ± 3.20	2.22 ± 2.40	.006
0−4	67 (77.9%)	7 (58.3%)	60 (81.1%)	.041
5−7	15 (17.4%)	3 (25.0%)	12 (16.2%)	
8−9	3 (3.5%)	1 (8.3%)	2 (2.7%)	
≥10	1 (1.2%)	1 (8.3%)	0	
ELAPSS score	11.05 ± 6.74	16.50 ± 6.91	10.16 ± 6.33	.002
0−9	37 (43.0%)	2 (25.0%)	35 (47.3%)	.046
10−19	37 (43.0%)	6 (41.7%)	31 (41.9%)	
≥20	12 (14.0%)	4 (33.3%)	8 (10.8%)	

Abbreviations: ACA, anterior cerebral artery; CRstalk, aneurysm‐to‐pituitary stalk contrast ratio; ICA, internal carotid artery; MCA, middle cerebral artery; PC, posterior circulation; PComA, posterior communicating artery.

## 4 DISCUSSION

This study showed a significant correlation of CAWE with the risk of aneurysm rupture and growth. The aneurysm wall enhancement has been used as an imaging marker of instability in UIA, which may correlate with inflammation revealed from histological findings (Gariel et al., 2020; Larsen et al., 2018; Quan et al., 2019; Shimonaga et al., 2018; Vergouwen et al., 2019). A larger wall enhancement area was independently associated with symptomatic UIAs (Zhu et al., 2020). The specificity of CAWE for differentiating stable aneurysms from unstable aneurysms was higher than focal AWE (X. Wang, Zhu, et al., 2019). A greater area of AWE may suggest a greater degree of inflammation, and CAWE was a more reliable imaging marker of aneurysm instability than AWE.

The ISUIA study showed that the risk of rupture in UIAs smaller than 7 mm was low, and the role of HR‐VWI in evaluating aneurysms smaller than 7 mm remained unclear. In our study, 25.3% (21/83) aneurysms showed wall enhancement, and 9.6% (8/83) showed CAWE, which was similar to previous studies investigating small UIAs (Zhu et al., 2018; Zwarzany et al., 2021). Zhu et al. (2018) found that AWE was an independent predictor of ruptured aneurysms, and CAWE was significantly associated with ruptured aneurysms instead of AWE in aneurysms smaller than 7 mm. Zwarzany et al. (2021) showed that the PHASES score was an independent risk factor for AWE in UIAs smaller than 10 mm. We found a significant correlation between CAWE and PHASES and ELAPSS scores in small UIAs (<7 mm), although two scores of our study belong to the lower risk categories for aneurysm rupture and growth. Nearly half of patients with ruptured aneurysms would have been assigned into the low rupture risk class (five‐year rupture risk <1%) if they had been discovered before the rupture in a retrospective application of PHASES score to the multicenter cohort of subarachnoid hemorrhage (Sturiale et al., 2021).

The ISUIA study showed that the rupture risk of aneurysms at anterior cerebral artery/posterior communicating artery/posterior circulation was higher than those at the middle cerebral artery and ICA (Wiebers, 2003). In a cohort of 79 patients with predominantly small UIAs, the strongest determinant of AWE was aneurysm size, followed by posterior communicating artery or middle cerebral artery aneurysm location (Backes et al., 2018). In our study of UIA with smaller than 7 mm, the percentage of CAWE in the middle cerebral artery was higher than in the anterior cerebral artery/posterior communicating artery/posterior circulation, and ICA.

There are several different modalities for quantitative measurement of aneurysmal wall enhancement to avoid the subjectivity of the readers (Omodaka et al., 2016; Roa, Zanaty, Ishii, et al., 2020; G.‐X. Wang, Gong, et al., 2019). Roa et al. compared the sensitivity and specificity of three different quantitative modalities of AWE on HR‐VWI among scanners from different manufacturers. They found that CRstalk using maximal SI was the best predictor of aneurysm instability (Roa, Zanaty, Osorno‐Cruz, et al., 2020). In our study, the optimal cutoff value of CRstalk for CAWE was 0.5, with a sensitivity of 81.8% and a specificity of 96%. Roa et al. showed that the cutoff value of CRstalk for AWE was 0.60, with a sensitivity of 81.5% and a specificity of 61%. The value of CRstalk in small UIAs was lower than large UIAs (Roa, Zanaty, Ishii, et al., 2020). In our study, the cutoff value of CRstalk for CAWE was lower than that of previous studies, which may be that most of the aneurysms were smaller than 7 mm. Roa, Zanaty, Ishii, et al. (2020) showed that daily intake of Aspirin was significantly protective for UIAs, reducing the odds of CRstalk ≥0.6 by an average of 78%, which indicated that CRstalk was a marker of inflammatory changes of the aneurysm wall.

The degree of vessel wall enhancement of UIAs using quantitative measurement was correlated with symptomatic and evolving aneurysms (Roa, Zanaty, Osorno‐Cruz, et al., 2020; G.‐X. Wang, Gong, et al., 2019; G.‐X. Wang et al., 2018). The wall enhancement index increased with increasing aneurysm size, PHASES score, and ELAPSS score (Zhang et al., 2020). Roa et al. (2021) found that UIAs with CRstalk ≥0.6 were considered “enhancing.” Enhancing UIAs scored higher in PHASES and ELAPSS scores than nonenhancing UIAs. We found that CRstalk values increased significantly with increasing aneurysm size, PHASES score, and ELAPSS score, indicating that CRstalk for CAWE may be a useful imaging marker to predict instability of UIAs.

This study has some limitations. First, this study was a single‐center design. The subjects included were patients with UIA hospitalized in our hospital with selection bias. Second, the number of patients in this study was relatively small, and the percentage of CAWE was relatively low in small aneurysms, resulting in a bias for statistics analysis. Third, the correlation between qualitative AWE patterns and quantitative CRstalk assessment was not performed in our study. The quantitative assessment of AWE using the wall enhancement index may improve assessing UIA instability (Fu et al., 2021). The thrombosis of the large aneurysms in this study was not assessed. Giant thrombosed aneurysms more often have a thick aneurysm wall with vasa vasorum and show aneurysm wall enhancement on HR‐VWI. Finally, no follow‐up study was performed to confirm that CAWE was an imaging marker of aneurysm instability.

Our findings show that CAWE on HR‐VWI is a valuable imaging marker for intracranial aneurysm instability in small UIAs. CRstalk (using maximal signal intensity value) ≥0.5 may be associated with a higher risk of intracranial aneurysm rupture and growth.

## CONFLICT OF INTEREST

All authors have no conflict of interest to declare.

## AUTHOR CONTRIBUTIONS

Zhong‐Song Shi participated in the design of the study. All authors participated in the interpretation and collection of the data. Xiao‐Bing Wu wrote the initial manuscript. Zhong‐Song Shi revised the manuscript. All authors critically reviewed and edited the manuscript and approved the final version.

## Data Availability

The data that support the findings of this study are available from the corresponding author upon reasonable request.
